# Gene Set Enrichment Analysis Detected Immune Cell-Related Pathways Associated with Primary Sclerosing Cholangitis

**DOI:** 10.1155/2022/2371347

**Published:** 2022-08-26

**Authors:** Pan Luo, Lin Liu, Weikun Hou, Ke Xu, Peng Xu

**Affiliations:** Department of Joint Surgery, Honghui Hospital, Xi'an Jiaotong University, Xi'an, Shanxi 710054, China

## Abstract

**Aim:**

To explore various immune cell-related causal pathways for primary sclerosing cholangitis (PSC).

**Methods:**

Immune cell-related pathway association study was conducted via integrative analysis of PSC GWAS summary and five immune cell-related eQTL datasets. The GWAS summary data of PSC was driven from 4,796 PSC cases and 19,955 healthy controls. The eQTL datasets of CD4+ T cells, CD8+ T cells, B cells, natural killer cells (NK), monocytes, and peripheral blood cells (PB) were collected from recently eQTL study. The PSC GWAS summary dataset was first aligned with eQTL datasets of six blood cells to obtain the GWAS summary data at overlapped eQTL loci, separately. For each type of cell, the obtained PSC GWAS summary dataset of eQTLs was subjected to pathway enrichment analysis. 853 biological pathways from Kyoto Encyclopedia of Genes and Genomes, BioCarta, and Reactome pathway databases were analyzed.

**Results:**

We identified 36 pathways for B cells, 33 pathways for CD4+ T cells, 28 pathways for CD8+ T cells, 33 pathways for monocytes (MN), 35 pathways for NK cells, and 33 for PB cells (all empirical *P* values <5.0 × 10^−5^). Comparing the pathway analysis results detected 25 pathways shared by five immune cells, such as KEGG_CELL_ADHESION_MOLECULES_CAMS (*P* value <5.0 × 10^−5^) and REACTOME_MHC_CLASS_II_ANTIGEN_ PRESENTATION (*P* value <5.0 × 10^−5^). Several cell-specific pathways were also identified, including BIOCARTA_INFLAM_PATHWAY (*P* value <5 × 10^−5^) for B cell.

**Conclusion:**

Our study holds potential to identify novel candidate causal pathways and provides clues for revealing the complex genetic mechanism of PSC.

## 1. Introduction

Primary sclerosing cholangitis (PSC) is a chronic cholestatic liver disease, characterized by widespread inflammation and fibrosis of bile ducts [1]. PSC is a rare disease with a higher incidence rate in men compared to women, which is capable of causing hepatic cirrhosis and subsequent liver failure. Recent study reported a prevalence of 4.03 PSC cases per 100,000 subjects and an incidence of 0.41 PSC per 100,000 person-years in a Caucasian population [2]. Additionally, up to 80% of PSC patients simultaneously suffer inflammatory bowel disease (IBD) [3] and an increased incidence of malignancy, such as intrahepatic cholangiocarcinoma, hepatobiliary cancer, and colorectal cancer [4–7]. Liver transplantation is the most definitive way for the treatment of PSC as there is no other better proven therapy of PSC to date.

Both environmental and genetic factors contribute to the development of PSC [8–10]. Genome-wide association studies (GWAS) have identified several susceptibility genes implicated in the development of PSC, such as FUT2 and FOXP1 [10–13]. Recently, the largest GWAS study of PSC to date reported four new significant loci and suggested that the decreased expression of UBASH3A was associated with a reduced risk of PSC [11]. Additionally, MMP-2 and RSPO3 have been identified to be associated with PSC [14, 15]. However, the genetic basis of PSC remains elusive now. The relationships of PSC with autoantibodies and the human leukocyte antigen (HLA) haplotypes suggested the dysfunction of the immune system implicated in the pathogenesis of PSC [16–19]. Recent studies revealed different roles of various immune cells in the development of PSC. For example, Schoknecht et al. found that CD4+ T cells from PSC patients exhibited decreased apoptosis sensitivity but not CD8+ T cells in peripheral blood [20]. Lampinen et al. observed that PSC patients had increased levels of CXCR3-positive CD8+ T cells but fewer CD25-positive CD4+ T cells [21]. Some drugs are involved in different molecular mechanisms in different cells [22, 23]. Therefore, it is reasonable to infer that there were differences in the molecular mechanism of different immune cells implicating in the development of PSC. However, to the best of our knowledge, there is no systematical genetic study of PSC considering potential differences among various immune cells by now.

It is well known that the gene expression is under genetic control [24]. Extensive expression quantitative trait loci (eQTLs) have been identified to bridge a gap between the variations of gene expression levels and genetic variations. More importantly, researchers found that disease-associated loci identified by GWAS were significantly enriched in eQTLs [25]. Recently, integrative analysis of GWAS and gene expression regulation loci data for exploring disease susceptibility genes has aroused widespread concern [26]. For example, Zhu and his colleagues proposed a method called summary data-based Mendelian randomization (SMR), which regards genetic variants as instrumental variables to evaluate the association between gene expression and target traits via integrating GWAS and eQTL datasets [27]. Recent evidence indicates that diseases or trait-associated variants operated in a cell-type specific manner [28]. The gene expression and signal transduction mechanisms of different cells are also different in inflammatory pathological response [29, 30]. An existing epilepsy study suggested that an epilepsy-eQTL analysis was more likely to explore epilepsy-causing genes compared with normal hippocampal tissue eQTL analyses [25]. However, the eQTLs identified in whole blood are generally not enough to reveal the genetic mechanism and require an expanded survey of disease-related tissues and cells involved in the genetic mechanism of diseases. A large scale immune cell-related eQTL study was conducted and identified in five immune cells related eQTLs, including CD4+ T cells, CD8+ T cells, B cells, NK cells, and monocytes [31].

Over the past decades, GWAS has identified a large number of susceptibility genes related to complex traits and diseases. However, the genetic risks of human diseases, especially complex diseases, are usually determined by the joint effects of multiple susceptibility loci. Furthermore, most of risk alleles identified by GWAS have weak genetic effects. The power of GWAS for detecting these loci was limited. The statistical power can be significantly increased if functionally relevant genetic loci (for instance, belonging to the same biological pathway) could be grouped together for testing. To address this issue, GWAS-based pathway enrichment analysis was proposed and successfully applied in the genetic studies of complex diseases [32]. In this study, utilizing the PSC GWAS summary data and five immune cell-related eQTL datasets, we conducted a large scale immune cell-related eQTL pathway enrichment analysis. Integrating the biological information of immune cell related eQTLs into genetic studies may provide novel clues for revealing the genetic structures of PSC. Considering the potential differences in the mechanism of different immune cells, our aim is to identify PSC-related pathways, so as to intervene against these pathways to provide new research ideas for the understanding and treatment of PSC.

## 2. Materials and Methods

### 2.1. GWAS Summary Dataset of PSC

A recent large-scale GWAS summary data of PSC was used here [11]. Briefly, a total of 4,796 PSC cases and 19,955 healthy controls from UK, US, Scandinavia, and Germany were enrolled in this study. PSC was diagnosed according to standard clinical, biochemical, cholangiographic, and histological criteria [33]. The patients with secondary sclerosing cholangitis were excluded. PSC cases and healthy controls from Scandinavia and Germany were genotyped using the Affymetrix Genome-Wide Human SNP Array 6.0 (Affymetrix), while the UK and US GWAS samples were genotyped using the Illumina HumanOmni2.5-8 BeadChip (Illumina). The SNPs with Hardy-Weinberg equilibrium (HWE) testing *P* values <1.0 × 10^−6^ in controls (excluding those in the HLA region) or call rate < 80% were removed. The samples genotyped by the Affy6 and Omni2.5 arrays were phased and imputed, separately. Prephasing was performed using the SHAPEIT2, and imputation was conducted using IMPUTE2 [34, 35]. A combined reference panel of the 1000 Genomes Phase 1 integrating version 3 and the UK10K cohort was used here, which involved 4,873 individuals and 42,359,694 SNPs. After quality control, 7,891,602 SNPs for 2,871 PSC cases and 12,019 healthy controls were analyzed. Genome-wide association tests were conducted by a linear mixed model accounting for population stratification [36]. Details about study subjects, experimental designs, quality control, and statistical analysis are available in the published study [11].

### 2.2. Cell-Specific eQTL Dataset

The eQTL datasets of CD4+ T cells, CD8+ T cells, B cells, NK cells, monocytes, and PB cells were driven from Ishigaki et al.'s study [31]. Briefly, PB cells were collected from 105 healthy volunteers (including 21 males and 84 females). The cell populations of CD4+ T cells, CD8+ T cells, B cells, NK cells, monocytes, and PB cells were sorted by FACS (Moflo XDP) followed a certain gating strategy. Total RNA from each cell subset was isolated using AllPrep DNA/RNA/miRNA Universal Kits (Qiagen) while the whole blood genomic DNA and total RNA were isolated using QIAamp DNA Blood Midi Kits and PAXgene Blood RNA Systems (Qiagen). Although microarray analysis has some advantages [37], one of the advantages of RNA sequencing is that it can quantify splice isomers comprehensively [31].

Genome-wide SNP genotyping for eQTL analysis was conducted using Infinium Omni Express Exome Bead Chips (Illumina). Quality control (QC) of the genotyping data was performed using PLINK 1.90 [38]. After quality control, 563,436 SNPs were prephased using SHAPEIT and imputed using IMPUTE2 against 1000 Genomes Phase 1 reference panel [39, 40]. RNA sequencing was performed using HiSeq 2500 platform. PEER was used to normalize the gene expression matrix [41]. Finally, the sample sizes for CD4+ T cells, CD8+ T cells, B cells, NK cells, monocytes, and PB were 103, 103, 104, 104, 105, and 98, respectively. Specific for this study, the identified gene-level eQTL datasets of six blood cells were used. Detailed information of the eQTL study was available in the published study [31].

### 2.3. Statistical Analysis

The PSC GWAS summary dataset was first aligned with the eQTL datasets of six blood cells to obtain the GWAS summary data at the overlapped eQTL SNP loci, separately. For each type of blood cell, the obtained PSC GWAS summary dataset of eQTLs was subjected to pathway enrichment analysis [42, 43]. The gene-pathway annotation datasets of Kyoto Encyclopedia of Genes and Genomes (KEGG), BioCarta, and Reactome were obtained from the GSEA Molecular Signatures Database (msigdb.v5.1) (http://software.broadinstitute.org/gsea/msigdb/index.jsp) [44]. The application of GSEA can provide good biological insights, including finding genes or regulators that participate in the same biological activity or pathway, sharing interaction genes or regulators, common cell compartmentalization, or association with disease [44, 45].853 biological pathways were analyzed in this study. A permutation procedure was used to calculate the empirical *P* value and false discovery rate (FDR) of each pathway for each cell. 20,000 permutations were conducted. Detailed analyzing procedures have been detailed in our previous studies [46]. For this study, the significant pathways were identified at empirical *P* value <5 × 10^−5^.

## 3. Results

After aligning the PSC GWAS summary and eQTL datasets, we used 37,080 eQTL variants for CD4^+^ T cells, 37,222 eQTL variants for CD8^+^ T cells, 51,798 eQTL variants for B cells, 44,886 eQTL variants for NK cells, 62,315 eQTL variants for MN cells, and 54,980 eQTL variants for PB cells. Pathway enrichment analysis identified 36 pathways for B cells, 33 pathways for CD4^+^ T cells, 28 pathways for CD8^+^ T cells, 33 pathways for monocytes, 35 pathways for NK cells, and 33 pathways for PB cells (all empirical *P* values <5.0 × 10^−5^).

**Figure 1:**
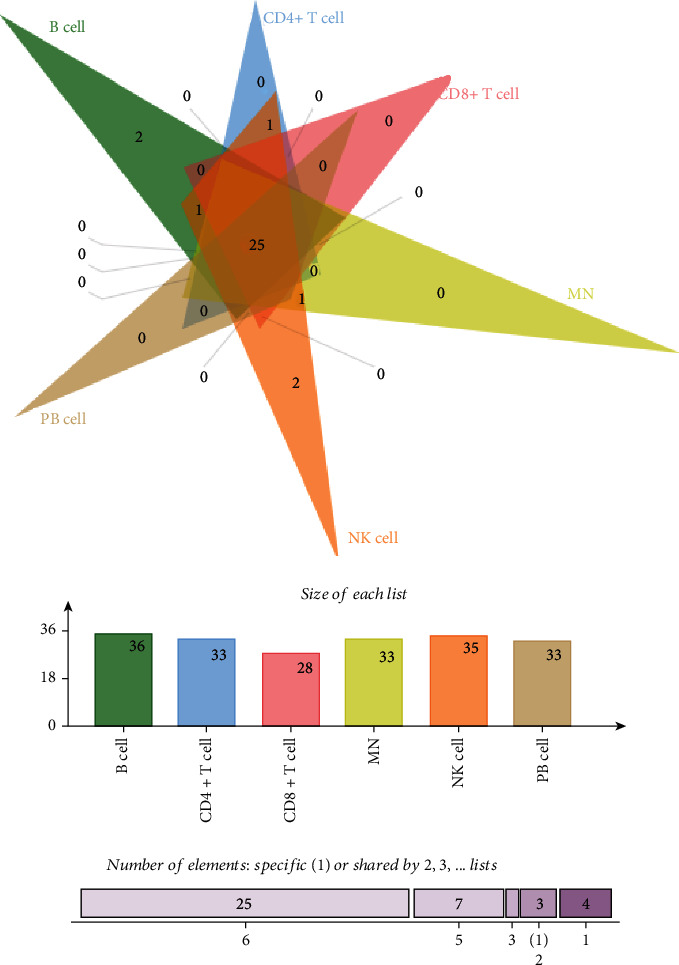
The number of significant pathways in five immune cells. As we can see, there are 36, 33, 28, 33, 35, and 33 significant pathways for B cells, CD4^+^ cells, CD8^+^ cells, monocytes, NK cells, and PB cells, separately. Among them, there are 25 common pathways shared by five immune cells and PB cell. Two pathways were only enriched in B cells and NK cells.

Further comparing the list of identified pathways, we detected 25 pathways significantly enriched in the eQTLs of five immune cells, such as KEGG_CELL_ADHESION_MOLECULES_CAMS (*P* value <5 × 10^−5^) and REACTOME_MHC_CLASS_II_ANTIGEN_ PRESENTATION (*P* value <5 × 10^−5^) (Table 1). Additionally, we also observed several cell-specific pathways (Figure 1). For instance, BIOCARTA_INFLAM_PATHWAY (*P* value <5 × 10^−5^) was highly enriched only in the eQTLs of B cell. The REACTOME_RNA_POL_I_RNA_POL_III_AND_ MITOCHONDRIAL_TRANSCRIPTION (*P* value <5 × 10^−5^) and REACTOME_ MEIOTIC_SYNAPSIS (*P* value <5 × 10^−5^) were only detected for NK cells. REACTOME_MEIOSIS was found to be enriched in the eQTLs of B cells (*P* value <5 × 10^−5^) and NK cells (*P* value <5 × 10^−5^).

## 4. Discussion

Integrative analysis of GWAS and eQTL datasets is capable of providing new insights into the genetic mechanism of complex diseases [27]. In this study, based on the latest large scale GWAS of PSC and eQTL studies, we conducted an eQTL pathway association analysis for five immune cells and PB cells. We detected a group of PSC-related biological pathways with common effects or cell type-related effects across various immune cells. We explored the potential roles of different immune cells in the pathogenesis of PSC through focusing on the genetic effects of eQTL pathways of different immune cells, which distinguished from previous studies. Our study results provide new clues for understanding the complex genetic mechanism of PSC.

This study detected multiple pathways with common causal effects across the five immune cells. For instance, KEGG_CELL_ADHESION_MOLECULES_CAMS was shared by all five immune cells. Cell adhesion molecules (CAMs) are proteins expressed on the cell surface and play a critical role in a variety of biologic processes, including the immune response and inflammation [47, 48]. Previous cell adhesion molecules study on PSC and primary biliary cirrhosis (PBC) found that the intercellular adhesion molecule-1 (ICAM-1) expression on biliary epithelium in PSC occurs mainly in late stage disease and therefore may be secondary to inflammation, whereas the ICAM-1 expression is less common in PBC [49]. Some genes of KEGG_CELL_ADHESION_MOLECULES_CAMS pathway have been suggested to be contributed to the development of PSC, such as CD86, CD28, and HLA complex. CD28 is an important immune regulatory protein expressed on T cells. It was identified that inflammatory CD28^−^ T cells accumulated in livers of patients with PSC. Moreover, activated CD28^−^ T cells released high levels of TNF and IFN proinflammatory cytokines and induced upregulation of intercellular cell adhesion molecule-1(CAM-1), HLA-DR, and CD40 by primary epithelial cells [50]. Another interesting gene in this pathway is CLDN1, which codes for protein claudin-1. Claudin-1 is a member of the claudin family, as well as a critical component of tight junction strands. Previous studies have demonstrated that CLDN1 mutation can cause ichthyosis-hypotrichosis-sclerosing-cholangitis (IHSC) [51, 52].

REACTOME_MHC_CLASS_II_ANTIGEN_PRESENTATION was another common pathway shared by the five immune cells. MHC class II molecules are a class of major histocompatibility complex (MHC) molecules normally found only on antigen-presenting cells, which serve a critical role in immune response, such as some endothelial cells and B cells. The MHC class II protein complex is encoded by the human leukocyte antigen gene complex (HLA) in human. PSC is strongly associated with the HLA complex, for instance, HLA-DRB1. Varies of HLA-DRB1 genes has been proved to be associated with PSC, including risk alleles and protective alleles [53, 54]. REACTOME_ANTIGEN_PRESENTATION_FOLDING_ASSEMBLY_AND_PEPTIDE_LOADING_OF_CLASS_I_MHC was also shared by the five immune cells. It consists of 21 genes, mainly involved in antigen presentation, including folding, assembly, and peptide loading of class I MHC. Antigen processing and presentation are key features of the immune response. Our study results support the implication of the immune system in the pathogenesis PSC, which is in line with what is known from previous studies [19, 55, 56].

We found that some biological pathways were associated with PSC in certain immune cells. For instance, the BIOCARTA_INFLAM_PATHWAY was significantly enriched in the eQTLs of B cells. BIOCARTA_INFLAM_PATHWAY consists of 29 genes, functionally involved in cytokines and inflammatory response. Some genes of BIOCARTA_INFLAM_PATHWAY have been demonstrated to contribute to the development of PSC. For instance, IL-8 in bile and serum has been identified as an important indicator of disease severity and prognosis for primary sclerosing cholangitis [57]. TGF-*β*1 has previously been shown to be associated with hepatic fibrosis [58]. Another study indicated that the menin/miR-24 axis can be modulated by altering the TGF-*β*1 expression to slow the progression of hepatic fibrosis into cirrhosis in PSC patients [59]. Recent studies demonstrated that dysfunction of the immune system was implicated in the development of PSC [60]. However, the biological effects of different immune cells in the pathogenesis of PSC remain unclear. Besides the common pathways shared by five analyzed immune cells, we also detected several immune cell specific pathways for PSC, supporting the different roles of immune cells in the development of PSC. Further biological studies are warranted to confirm our findings.

One advantage of this study is that we focused on the gene expression rather than genetic variations or SNPs. Although a dramatic increase in the number of reported SNPs implicating in a wide variety of diseases has been reported recently, the majority of them were located in the noncoding chromosomal regions. Compared with GWAS-based pathway association analysis, eQTL-based pathway analysis can help to identify novel causal genes and pathways implicated in PSC pathogenesis through gene expression regulation. Furthermore, the discovery of identified eQTL pathways varied across cell types demonstrated that different immune cells may play different roles in the pathogenesis of PSC, providing novel clues for revealing the complex genetic basis of PSC and it might be a new therapeutic target for PSC.

There is an issue in our study that should be noted. The population of PSC GWAS summary dataset was from UK, US, Scandinavia, and Germany other than Chinese, which lowers the priority of our work in some extent. Further biological studies in Chinese population are needed.

## 5. Conclusion

In conclusion, we conducted an immune cell-related eQTL-based pathway association study of PSC through integrating PSC GWAS and eQTL datasets. We identified a group of PSC-related biological pathways with common effects or cell specific effects across various immune cells. Our study holds potential to identify novel candidate causal pathways and provides novel clues for revealing the complex genetic mechanism of PSC.

## Figures and Tables

**Table 1:** List of identified pathways with empirical *P* value <5 × 10^−5^ in five immune cells and PB cells.

Pathway	B cells	CD4^+^ cells	CD8^+^ cells	MN cells	NK cells	PB cells
KEGG_ALLOGRAFT_REJECTION	**<5E-05**	**<5E-05**	**<5E-05**	**<5E-05**	**<5E-05**	**<5E-05**
KEGG_ANTIGEN_PROCESSING_AND_PRESENTATION	**<5E-05**	**<5E-05**	**<5E-05**	**<5E-05**	**<5E-05**	**<5E-05**
KEGG_ASTHMA	**<5E-05**	**<5E-05**	**<5E-05**	**<5E-05**	**<5E-05**	**<5E-05**
KEGG_AUTOIMMUNE_THYROID_DISEASE	**<5E-05**	**<5E-05**	**<5E-05**	**<5E-05**	**<5E-05**	**<5E-05**
KEGG_CELL_ADHESION_MOLECULES_CAMS	**<5E-05**	**<5E-05**	**<5E-05**	**<5E-05**	**<5E-05**	**<5E-05**
KEGG_GRAFT_VERSUS_HOST_DISEASE	**<5E-05**	**<5E-05**	**<5E-05**	**<5E-05**	**<5E-05**	**<5E-05**
KEGG_INTESTINAL_IMMUNE_NETWORK_FOR_IGA_PRODUCTION	**<5E-05**	**<5E-05**	**<5E-05**	**<5E-05**	**<5E-05**	**<5E-05**
KEGG_LEISHMANIA_INFECTION	**<5E-05**	**<5E-05**	**<5E-05**	**<5E-05**	**<5E-05**	**<5E-05**
KEGG_SYSTEMIC_LUPUS_ERYTHEMATOSUS	**<5E-05**	**<5E-05**	**<5E-05**	**<5E-05**	**<5E-05**	**<5E-05**
KEGG_TYPE_I_DIABETES_MELLITUS	**<5E-05**	**<5E-05**	**<5E-05**	**<5E-05**	**<5E-05**	**<5E-05**
KEGG_VIRAL_MYOCARDITIS	**<5E-05**	**<5E-05**	**<5E-05**	**<5E-05**	**<5E-05**	**<5E-05**
REACTOME_ANTIGEN_PRESENTATION_FOLDING_ASSEMBLY_AND_PEPTIDE_LOADING_OF_CLASS_I_MHC	**<5E-05**	**<5E-05**	**<5E-05**	**<5E-05**	**<5E-05**	**<5E-05**
REACTOME_ANTIGEN_PROCESSING_CROSS_PRESENTATION	**<5E-05**	**<5E-05**	**<5E-05**	**<5E-05**	**<5E-05**	**<5E-05**
REACTOME_DOWNSTREAM_TCR_SIGNALING	**<5E-05**	**<5E-05**	**<5E-05**	**<5E-05**	**<5E-05**	**<5E-05**
REACTOME_ENDOSOMAL_VACUOLAR_PATHWAY	**<5E-05**	**<5E-05**	**<5E-05**	**<5E-05**	**<5E-05**	**<5E-05**
REACTOME_ER_PHAGOSOME_PATHWAY	**<5E-05**	**<5E-05**	**<5E-05**	**<5E-05**	**<5E-05**	**<5E-05**
REACTOME_GENERATION_OF_SECOND_MESSENGER_MOLECULES	**<5E-05**	**<5E-05**	**<5E-05**	**<5E-05**	**<5E-05**	**<5E-05**
REACTOME_INTERFERON_GAMMA_SIGNALING	**<5E-05**	**<5E-05**	**<5E-05**	**<5E-05**	**<5E-05**	**<5E-05**
REACTOME_INTERFERON_SIGNALING	**<5E-05**	**<5E-05**	**<5E-05**	**<5E-05**	**<5E-05**	**<5E-05**
REACTOME_MHC_CLASS_II_ANTIGEN_PRESENTATION	**<5E-05**	**<5E-05**	**<5E-05**	**<5E-05**	**<5E-05**	**<5E-05**
REACTOME_PD1_SIGNALING	**<5E-05**	**<5E-05**	**<5E-05**	**<5E-05**	**<5E-05**	**<5E-05**
REACTOME_PHOSPHORYLATION_OF_CD3_AND_TCR_ZETA_CHAINS	**<5E-05**	**<5E-05**	**<5E-05**	**<5E-05**	**<5E-05**	**<5E-05**
REACTOME_RNA_POL_I_PROMOTER_OPENING	**<5E-05**	**<5E-05**	**<5E-05**	**<5E-05**	**<5E-05**	**<5E-05**
REACTOME_TCR_SIGNALING	**<5E-05**	**<5E-05**	**<5E-05**	**<5E-05**	**<5E-05**	**<5E-05**
REACTOME_TRANSLOCATION_OF_ZAP_70_TO_IMMUNOLOGICAL_SYNAPSE	**<5E-05**	**<5E-05**	**<5E-05**	**<5E-05**	**<5E-05**	**<5E-05**
BIOCARTA_INFLAM_PATHWAY	**<5E-05**	*0.0064*	*0.0149*	*0.0539*	*0.001*	*0.0724*
KEGG_NATURAL_KILLER_CELL_MEDIATED_CYTOTOXICITY	**<5E-05**	*1E-04*	*0.0014*	*0.0011*	*0.001*	*0.0003*
REACTOME_MEIOTIC_SYNAPSIS	*0.0015*	*1E-04*	*0.0254*	*0.002*	**<5E-05**	*1E-04*
REACTOME_RNA_POL_I_RNA_POL_III_AND_MITOCHONDRIAL_TRANSCRIPTION	*0.0002*	*0.0014*	*0.0054*	*1E-04*	**<5E-05**	*0.0001*
REACTOME_MEIOSIS	**<5E-05**	*0.0002*	*0.0029*	*0.0015*	**<5E-05**	*1E-04*
REACTOME_TELOMERE_MAINTENANCE	*0.002*	**<5E-05**	*0.0934*	*0.004*	**<5E-05**	*0.001*
REACTOME_DEPOSITION_OF_NEW_CENPA_CONTAINING_NUCLEOSOMES_AT_THE_CENTROMERE	*0.0001*	*0.0001*	*0.0019*	**<5E-05**	**<5E-05**	*1E-04*
REACTOME_COSTIMULATION_BY_THE_CD28_FAMILY	**<5E-05**	*0.0001*	**<5E-05**	**<5E-05**	**<5E-05**	**<5E-05**
REACTOME_IMMUNOREGULATORY_INTERACTIONS_BETWEEN_A_LYMPHOID_AND_A_NON_LYMPHOID_CELL	**<5E-05**	**<5E-05**	*0.0034*	*0.0002*	*0.0044*	**<5E-05**
REACTOME_AMYLOIDS	**<5E-05**	**<5E-05**	*0.0006*	**<5E-05**	**<5E-05**	**<5E-05**
REACTOME_MEIOTIC_RECOMBINATION	**<5E-05**	**<5E-05**	*0.0004*	**<5E-05**	**<5E-05**	**<5E-05**
REACTOME_PACKAGING_OF_TELOMERE_ENDS	**<5E-05**	**<5E-05**	*0.0049*	**<5E-05**	**<5E-05**	**<5E-05**
REACTOME_RNA_POL_I_TRANSCRIPTION	**<5E-05**	**<5E-05**	*0.0034*	**<5E-05**	**<5E-05**	**<5E-05**
REACTOME_CYTOKINE_SIGNALING_IN_IMMUNE_SYSTEM	**<5E-05**	**<5E-05**	**<5E-05**	**<5E-05**	*0.0002*	**<5E-05**
REACTOME_INTERFERON_ALPHA_BETA_SIGNALING	**<5E-05**	**<5E-05**	**<5E-05**	**<5E-05**	*0.0034*	**<5E-05**

Bold numbers represents the empirical *P* value ≤5 × 10^−5^ in these six cells. Italic numbers represents the empirical *P* value >5 × 10^−5^ in these six cells.

## Data Availability

Data used in the present study are all publicly available. Authors will provide the data upon reasonable request.

## Abbreviations

PSC: Primary sclerosing cholangitis
NK: Natural killer
MN: Monocyte
GWAS: Genome-wide association study
eQTLs: Expression quantitative trait loci
KEGG: Kyoto Encyclopedia of Genes and Genomes
HLA: Human leukocyte antigen gene complex
CAM: Cell adhesion molecules
IHSC: Ichthyosis-hypotrichosis-sclerosing-cholangitis
SMR: Summary data-based Mendelian randomization
MHC: Major histocompatibility complex
GSEA: Gene set enrichment analysis.
